# From inflammation to rupture: advances in the pathogenesis of pseudoaneurysm formation in Vascular Behçet’s Disease

**DOI:** 10.3389/fimmu.2026.1930449

**Published:** 2026-08-20

**Authors:** Yanfei Yang, Yuexin Chen

**Affiliations:** Department of Vascular Surgery, Peking Union Medical College Hospital, Chinese Academy of Medical Sciences & Peking Union Medical College, Beijing, China

**Keywords:** immunopathology, neutrophil extracellular traps, THBS1^high^ macrophages, VBD pseudoaneurysm, VSMC phenotypic switching

## Abstract

**Purpose of review:**

This narrative review aims to comprehensively synthesize recent advances in the immunopathological mechanisms and genetic landscape underlying pseudoaneurysm formation in Vascular Behçet’s Disease (VBD). We integrate key discoveries from 2010 to 2026 regarding genetic susceptibility, immune cell dysregulation, inflammatory mediator networks, and structural deterioration of the vascular wall. Unlike previous reviews that primarily focused on clinical manifestations and descriptive observations, this article proposes a multilevel pathogenic cascade model linking genetic predisposition to full-thickness vascular wall destruction. We critically evaluate current consensus and controversies, identify unresolved knowledge gaps, and highlight potential strategies for early risk stratification and precision intervention. These insights may provide a theoretical framework for addressing the clinical challenges associated with the high rupture risk and recurrence rate of VBD-associated pseudoaneurysms.

**Recent findings:**

At the genetic level, the HLA-B51/ERAP1 axis establishes a fundamental susceptibility background, while non-HLA genes, including TNFAIP3, IL10, and CCR1, contribute to fine-tuning immune regulatory thresholds. At the cellular level, excessive neutrophil NETosis, activation of THBS1^high^ macrophages, aberrant Th17 responses, and disruption of the Treg/effector T-cell balance collectively form a self-amplifying inflammatory network. Notably, THBS1^high^ macrophages have been reported to provide single-cell-level evidence linking immune activation to aneurysm development by driving vascular smooth muscle cell (VSMC) phenotypic switching; however, this finding derives from a single-center exploratory study and awaits independent replication. At the inflammatory mediator level, TNF-α functions as a central driver that cooperates with matrix metalloproteinase (MMP)-mediated extracellular matrix degradation, JAK/STAT signaling, and the IL-23/IL-17 axis to establish a highly interconnected pro-inflammatory signaling network. At the vascular structural level, inflammation propagates from the adventitial vasa vasorum toward the inner vascular layers, accompanied by medial elastic fiber disruption and VSMC phenotypic transition, ultimately resulting in pseudoaneurysm formation. The YAP/TAZ mechanotransduction pathway further reinforces a vicious cycle between vascular expansion and inflammation. Current animal models, including herpes simplex virus (HSV)-induced models and HLA-B51 transgenic models, fail to consistently reproduce vascular phenotypes, representing a major barrier to mechanistic investigation.

**Summary:**

VBD-associated pseudoaneurysm represents a multilevel pathogenic cascade driven by the interaction of genetic susceptibility, immune cell amplification, inflammatory mediator networks, and progressive vascular wall degeneration. Integrated stratification based on immunological and genetic biomarkers may enable early identification of high-risk patients and facilitate individualized therapeutic strategies. Targeting the THBS1–macrophage axis and the NETs–IL-17A inflammatory circuit may represent a promising direction for precision intervention, although substantial translational barriers remain. Future studies should prioritize the development of vascular phenotype–relevant animal models, conduct dedicated genetic investigations focused on pseudoaneurysm susceptibility, and integrate multi-omics approaches to bridge mechanistic discoveries with clinical translation.

## 1 Introduction

Behçet disease (BD) is a systemic vasculitis characterized by recurrent oral and genital ulcers, ocular inflammation, and mucocutaneous lesions, with a particularly high prevalence along the historical Silk Road region. Although its etiology remains incompletely understood, the prevailing hypothesis suggests that genetically predisposed individuals develop dysregulated immune responses following exposure to environmental triggers, including infectious stimuli and microbiome perturbations, ultimately leading to systemic vascular inflammation (1, 2). When BD involves medium- and large-sized vessels, it is classified as vascular Behçet’s disease (VBD), which represents the clinical subtype associated with the poorest prognosis. The vascular manifestations of VBD are highly heterogeneous: arterial involvement is predominantly characterized by pseudoaneurysm formation, true aneurysms, and arterial occlusion, whereas venous manifestations mainly include deep vein thrombosis and superficial thrombophlebitis (3, 4).

Among arterial complications of VBD, pseudoaneurysms have attracted particular attention because of their high risk of rupture and mortality. Unlike atherosclerotic aneurysms, which develop primarily through lipid accumulation and progressive intimal degeneration, VBD-associated pseudoaneurysms arise from transmural vascular inflammation that destroys the structural integrity of the entire vessel wall, followed by blood extravasation and encapsulation by surrounding tissues, resulting in the formation of a pulsatile hematoma. This fundamentally distinct, non-atherosclerotic inflammatory mechanism accounts for the unique clinical characteristics of VBD pseudoaneurysms, including younger age at onset, rapid progression, multifocal involvement, and a markedly elevated risk of rupture (5, 6).

The clinical burden of VBD-associated pseudoaneurysms remains substantial. In untreated cases, the reported rupture rate exceeds 60%, with mortality after rupture ranging from 50% to 70%. Even after surgical or endovascular repair, recurrent aneurysm formation or anastomotic pseudoaneurysms develop in approximately 30–40% of patients within 5 years (7, 8). The high postoperative recurrence rate indicates that current surgical and interventional approaches primarily address the mechanical consequences of vascular wall disruption but fail to eliminate the underlying immunological drivers of persistent vascular inflammation.

In recent years, the emergence of advanced technologies, including single-cell RNA sequencing, whole-exome sequencing, spatial transcriptomics, and multiplex immunofluorescence, has enabled unprecedented resolution in dissecting the molecular and cellular mechanisms underlying VBD-associated pseudoaneurysm formation (9–11). In this narrative review, we comprehensively synthesize advances reported between 2010 and 2026, examining the pathogenesis of VBD pseudoaneurysms across multiple biological levels, including genetic susceptibility, immune cell dysregulation, inflammatory mediator networks, vascular wall remodeling, and experimental models. Furthermore, we propose an integrated multilevel pathogenic framework that connects genetic predisposition with immune amplification and progressive vascular wall destruction.

Search strategy and selection criteria: This is a narrative review based on a structured literature search of PubMed, Embase, and Web of Science, supplemented by hand-searching of reference lists. Searches were performed without language restriction and covered the period from January 2010 to June 2026, with older foundational studies included where mechanistically relevant. Search terms combined Medical Subject Headings and free-text terms including “Behçet disease” or “Behçet syndrome”, “vascular Behçet”, “pseudoaneurysm” or “aneurysm”, “immunopathology”, “single-cell”, “genetics”, “HLA-B51”, “neutrophil extracellular traps”, “macrophage”, “vascular smooth muscle cell”, and “animal model”. Original research articles, systematic reviews, and meta-analyses reporting on pathogenic mechanisms of VBD-associated pseudoaneurysms or related vascular inflammation were prioritized; case reports were used only for illustrative clinical context. Because this is a narrative synthesis rather than a systematic review, no formal risk-of-bias assessment or PRISMA flow was applied, and the cited literature should be interpreted as a selected rather than exhaustive evidence base. Evidence from different sources—human VBD tissue, non-vascular BD studies, other aneurysm models (e.g., abdominal aortic aneurysm, aortic dissection), animal experiments, and bioinformatic predictions—is distinguished in the text and in **Table 1**, as these sources carry different levels of directness and certainty.

**Table 1:** Pathological mechanisms of vascular wall structural destruction in VBD pseudoaneurysms.

Pathological link	Key molecules/cells	Pathological effects	Evidence source/study type	Key limitations	Refs
Vasa vasorum inflammation	Adventitial microvessels/infiltrating immune cells	Wall ischemia; inflammation spreading from outside inward	Human VBD tissue + other aneurysm models (AAA/dissection)	Mostly indirect; borrowed from non-VBD models	(54, 56)
Medial elastic fiber disruption	MMP-2/MMP-9/elastase	Loss of elastic recoil; vessel dilatation	Human VBD tissue + AAA literature	Associative; small samples	(5, 9, 42)
Smooth muscle cell apoptosis	TNF-α/IL-1β/oxidative stress	Medial thinning; loss of active tension	Human VBD tissue + other aneurysm models	Cross-sectional; confounding by activity	(5, 9)
VSMC phenotypic switching	THBS1/YAP-TAZ/BAF60c	Contractile → synthetic phenotype; MMP secretion	Single-center scRNA-seq (VBD) + animal/AAA data	Single-center; not independently replicated	(9, 44, 57)
Full-thickness penetration and pseudoaneurysm	Inflammation breaks through adventitia	Blood extravasation; pulsatile hematoma	Human VBD tissue	End-stage tissue; selection bias	(5, 9, 15)
Postoperative anastomotic pseudoaneurysm	Persistent inflammation + suture dehiscence	Poor healing at anastomosis; high recurrence rate	Clinical observational studies	Retrospective; heterogeneous definitions	(7, 8, 15)

## 2 Overview of vascular Behçet’s disease–associated pseudoaneurysms

### 2.1 Epidemiological characteristics

The prevalence of vascular involvement in Behçet disease (BD) varies considerably across geographic regions and populations, with reported frequencies ranging from 5% to 40%. Among these manifestations, arterial involvement accounts for approximately 2.2–17% of cases. Male sex, young age at disease onset (<25 years), and HLA-B51 positivity represent major risk factors associated with vascular involvement (3, 12).

A retrospective study from China including 166 patients with vascular Behçet’s disease (VBD) provided detailed anatomical distribution data of arterial lesions. Arterial involvement predominantly affected the abdominal aorta (31%), femoral arteries (23%), iliac arteries (15%), and carotid arteries (10%), with pseudoaneurysms accounting for 35.5% of all arterial lesions (12). A 2026 cohort study from Qatar further demonstrated that 26.5% of VBD patients exhibited simultaneous arterial and venous involvement, highlighting the systemic and diffuse impact of this disease on the vascular system (8).

From an epidemiological perspective, VBD-associated pseudoaneurysms predominantly occur in males aged 30–45 years, with a mean age at diagnosis of approximately 35 years. Multifocal aneurysmal involvement is observed in 18–30% of patients, in marked contrast to conventional atherosclerotic aneurysms (7). Rupture of aortic pseudoaneurysms may result in catastrophic circulatory collapse, whereas rupture of pulmonary artery pseudoaneurysms often presents as life-threatening massive hemoptysis, with emergency mortality rates reaching up to 80%. With appropriate medical and interventional management, the 5-year survival rate of patients with VBD-associated pseudoaneurysms is approximately 70–85%; however, postoperative recurrence and development of new aneurysmal lesions remain major determinants of long-term outcomes (13, 14).

### 2.2 Clinicopathological features

The clinical manifestations of VBD-associated pseudoaneurysms vary according to anatomical location, lesion size, and rupture status. Pain represents the most common initial symptom, reported in approximately 60–80% of patients. A pulsatile mass is the most characteristic clinical finding and can be detected in 50–70% of cases, often accompanied by thrill and vascular bruits.

Approximately 20–30% of patients present with symptoms caused by compression of adjacent structures. For example, carotid artery pseudoaneurysms may compress the trachea, leading to dyspnea and hoarseness; abdominal aortic lesions may compress the duodenum, causing abdominal distension and vomiting; and femoral artery lesions may compress the femoral nerve, resulting in lower limb sensory abnormalities. Additionally, 10–25% of patients initially present with rupture-related hemorrhage (3, 6).

Histologically, VBD-associated pseudoaneurysms differ fundamentally from atherosclerotic aneurysms. Gross examination typically reveals irregular and fragile aneurysmal walls, marked edema, and thickened hyperemic surrounding soft tissues. Histological analysis demonstrates transmural inflammatory infiltration, disruption and loss of medial elastic fibers, and dense adhesion between the adventitia and adjacent tissues.

Microscopically, characteristic features include fibrinoid necrosis of the intima accompanied by infiltration of neutrophils and lymphocytes, destruction of medial elastic fibers, depletion or loss of vascular smooth muscle cells (VSMCs), and adventitial vasa vasorum proliferation with perivascular inflammatory infiltration. The aneurysmal wall lacks the normal three-layered arterial architecture and is instead primarily composed of fibrous tissue, organized thrombus, and inflammatory granulation tissue (5, 15).

This extensive transmural inflammatory destruction represents the histological basis underlying the high rupture risk and recurrence rate of VBD-associated pseudoaneurysms and provides the pathological foundation for understanding subsequent immune-mediated mechanisms.

## 3 Genetic susceptibility underlying vascular Behçet’s disease

### 3.1 Genetic association of HLA-B51 and ERAP1

HLA-B51 represents the most established genetic susceptibility marker for Behçet disease. Across global cohorts, HLA-B51 positivity is observed in approximately 40–80% of BD patients, compared with 10–20% in healthy populations. Carriers exhibit an approximately 5–6-fold increased risk of developing BD, and HLA-B51 positivity is positively associated with vascular involvement (1, 16).

HLA-B51 belongs to the major histocompatibility complex class I (MHC-I) family and functions by presenting intracellular antigen-derived peptides to CD8^+^ T cells. One prevailing hypothesis proposes that altered antigen peptide repertoires presented by HLA-B51 lead to aberrant activation of CD8^+^ T cells, thereby initiating autoimmune attacks against vascular tissues. A 2022 study directly demonstrated that the HLA-B51/ERAP1-Hap10 combination reshapes human CD8^+^ T-cell immune responses, suggesting an epistatic interaction between these two genetic factors (17).

Endoplasmic reticulum aminopeptidase 1 (ERAP1) is responsible for trimming antigenic peptides to optimal lengths suitable for MHC-I binding. Genetic polymorphisms in ERAP1 can alter peptide-processing patterns and subsequently influence the repertoire of peptides presented by HLA-B51. Therefore, the HLA-B51–ERAP1 axis represents one of the major genetic mechanisms contributing to BD susceptibility (17, 18).

The European Alliance of Associations for Rheumatology (EULAR) has classified BD as an MHC-I-opathy, highlighting shared MHC-I-mediated immunopathological mechanisms with other diseases, including ankylosing spondylitis and acute anterior uveitis (18). However, HLA-B51 is not a disease-determining gene for VBD. Approximately 20–40% of BD patients are HLA-B51 negative, and many HLA-B51-positive individuals never develop disease, indicating that HLA-B51 provides a susceptibility background rather than a sufficient condition for disease onset. Environmental triggers and additional genetic factors are therefore required for disease manifestation.

### 3.2 Non-HLA susceptibility genes

Genome-wide association studies (GWAS) and whole-exome sequencing analyses have identified multiple non-HLA susceptibility loci associated with BD. Several genes appear to be particularly relevant to vascular involvement.

Loss-of-function mutations in TNFAIP3, which encodes the ubiquitin-editing protein A20, result in A20 haploinsufficiency and cause early-onset BD-like syndromes characterized by severe vascular inflammation (19). GWAS studies have identified CCR1 and IL10 loci as contributors to BD pathogenesis, potentially through regulation of macrophage-mediated inflammatory responses and promotion of an M1-dominant inflammatory phenotype (20). The MCP-1 (monocyte chemoattractant protein-1) promoter −2518 polymorphism has also been associated with susceptibility to VBD, potentially by enhancing monocyte recruitment to vascular tissues (21). In addition, ACE insertion/deletion polymorphisms may increase BD susceptibility through modulation of the renin–angiotensin system and vascular inflammatory responses (22).

A whole-exome sequencing study published in 2026 identified novel DNA sequence variants in patients with VBD, providing new insights into the genetic heterogeneity underlying vascular manifestations (23).

Overall, the genetic architecture of BD is highly polygenic. HLA-B51 establishes the primary susceptibility background, whereas ERAP1, TNFAIP3, IL10, CCR1, and other genes contribute to fine regulation of immune activation and inflammatory responses. Interactions among genetic variants, epistatic effects, and environmental factors collectively determine disease phenotypes, including whether vascular involvement occurs and the specific vascular manifestations that develop.

Currently, dedicated GWAS studies focusing specifically on pseudoaneurysm phenotypes are lacking, representing a critical knowledge gap and an important direction for future investigation.

## 4 Immune cell-mediated vascular inflammation

### 4.1 Neutrophils and NETosis

The contribution of neutrophils to Behçet disease extends far beyond their traditional role as innate immune effector cells. Neutrophils from BD patients exhibit a hyperresponsive phenotype, characterized by enhanced chemotaxis, phagocytosis, and degranulation even in the absence of external stimulation. This intrinsic hyperactivation of innate immune cells is considered a major driver of persistent and uncontrolled inflammation in BD (11, 24).

The discovery of neutrophil extracellular traps (NETs) has provided new insights into the mechanisms underlying vascular injury in BD. NETs are extracellular web-like structures composed of decondensed chromatin decorated with granular proteins, including neutrophil elastase, myeloperoxidase (MPO), and cathepsin G. Although NET formation represents an evolutionarily conserved mechanism designed to capture and eliminate pathogens, excessive NETosis under autoimmune inflammatory conditions can induce substantial tissue damage.

A pivotal study published in 2019 demonstrated significantly elevated circulating NET levels in BD patients, with NET abundance positively correlated with disease activity (25). Subsequent research in 2020 further revealed that NETs derived from BD patients promoted abnormal macrophage activation, establishing a NET–macrophage feed-forward loop that may contribute to sustained amplification of vascular inflammation (26). NETs are also closely associated with thrombosis. Increasing evidence suggests that thrombus formation in BD is not simply attributable to conventional hypercoagulability but rather represents an immune-mediated thrombotic process driven by NETs (immunothrombosis) (27).

Although this review focuses on arterial pseudoaneurysm formation, evidence from venous thrombosis and deep vein thrombosis (DVT) in BD is invoked here because the underlying NETs-mediated immunothrombotic mechanisms are shared across vascular beds. Arterial pseudoaneurysm and venous thrombosis are nevertheless distinct vascular phenotypes with divergent hemodynamic and structural contexts, and such mechanistic parallels should not be conflated with equivalence; venous-derived evidence is therefore interpreted as supportive rather than directly probative for arterial wall destruction.

Recent studies have further highlighted the heterogeneity of NET-producing neutrophil populations. A 2026 study identified a CD177^+^ neutrophil subset that promotes endothelial senescence through NET release, providing a more refined cellular explanation for vascular injury in VBD (28). Furthermore, the NETs–CD44–IL-17A feedback circuit proposed in 2025 established a mechanistic connection between neutrophil activation and Th17 immunity. NETs activate Th17 cells through CD44 signaling to induce IL-17A production, whereas IL-17A subsequently stimulates additional NET release from neutrophils, generating a self-sustaining inflammatory loop (29).

In addition, deficiency of the plasma-derived exosomal protein SHP2 has been shown to induce excessive neutrophil activation in BD, providing an extracellular signaling mechanism that contributes to neutrophil hyperresponsiveness (30).

### 4.2 Pathogenic differentiation of macrophages

The role of macrophages in VBD-associated pseudoaneurysm formation has been informed by a 2025 single-cell RNA sequencing study published in BMC Medicine; as this was a single-center exploratory investigation, its findings should be considered provisional pending independent replication. Using single-cell RNA sequencing analysis of VBD aneurysmal tissues, this study identified marked enrichment of THBS1”high macrophages within diseased vascular walls.

These macrophages not only produce abundant pro-inflammatory mediators but also secrete thrombospondin-1 (THBS1), which induces phenotypic switching of vascular smooth muscle cells (VSMCs) from a contractile phenotype toward a synthetic phenotype. Synthetic VSMCs acquire enhanced proliferative and secretory capabilities, producing increased levels of matrix metalloproteinases (MMPs) and inflammatory mediators. This pathological interaction between macrophages and VSMCs may ultimately promote aneurysm development (9).

This study provided single-cell-level evidence linking a specific macrophage population, VSMC phenotypic transition, and aneurysm formation, representing a potentially important advance in understanding VBD pathogenesis that nonetheless requires independent validation.

Before the identification of THBS1”high macrophages, macrophage studies in BD mainly focused on conventional M1/M2 polarization patterns. Genetic studies have demonstrated that BD-associated CCR1 and IL10 loci contribute to disease development by promoting macrophage-driven inflammatory responses dominated by M1-like polarization (20). Studies showing that curcumin exerts anti-inflammatory effects through modulation of M1/M2 macrophage polarization further support the importance of macrophage functional balance as a therapeutic target (31). Additionally, a C1q-high monocyte/macrophage precursor population has been implicated in BD-associated inflammation, potentially representing an abnormal differentiation trajectory toward pathogenic macrophage states (32).

### 4.3 Aberrant activation of T-cell subsets

T cells represent central mediators of adaptive immune responses in BD. Among CD4^+^ T-cell subsets, disruption of the Th1/Th17 balance is particularly prominent. Th17 cells and their signature cytokine IL-17A are significantly elevated in both peripheral blood and affected tissues of BD patients, with levels closely associated with disease activity (33, 34).

Th17 cells promote recruitment of neutrophils into target tissues through IL-17A secretion, whereas NETs released by activated neutrophils subsequently enhance Th17 activation. The previously described NETs–CD44–IL-17A circuit represents the molecular basis underlying this reciprocal amplification between innate and adaptive immunity (29).

The contribution of CD8^+^ T cells has also gained increasing attention. Specific antigen peptides presented by HLA-B51 may induce abnormal activation of CD8^+^ T cells, resulting in cytotoxic responses against vascular wall cells (17). Transcriptomic analyses of iris tissues have demonstrated enhanced LCK signaling and T-cell-mediated immune responses in BD-associated uveitis, suggesting that T-cell signaling pathways may represent potential therapeutic targets (35).

Conversely, reduced numbers or impaired suppressive function of regulatory T cells (Tregs) may weaken immune tolerance mechanisms, allowing persistent activation of autoreactive effector T cells and maintenance of chronic vascular inflammation (24).

### 4.4 Contribution of monocytes

As precursors of macrophages, monocytes have recently received renewed attention in the pathogenesis of BD. Transcriptomic analyses have revealed distinctive gene expression patterns in peripheral blood mononuclear cells from BD patients, with prominent alterations in pathways associated with neutrophil recruitment and inflammatory activation (11).

A 2021 study identified abnormal distribution of monocyte subsets in BD patients, suggesting that imbalance between classical and non-classical monocytes may reflect an increased tendency toward differentiation into inflammatory macrophages (32). More recently, a 2025 single-cell study identified a CCL3^+^ classical monocyte subset associated with autoimmune pathology in BD (36).

The MCP-1 promoter −2518 polymorphism may contribute to VBD development by enhancing monocyte chemotaxis toward vascular tissues (21). Furthermore, the clinical efficacy of leukocytapheresis, which removes activated circulating leukocytes, provides indirect evidence supporting the pathogenic importance of myeloid cells in BD-associated inflammation (37).

### 4.5 Immune-cell cooperation and self-amplifying inflammatory networks

The vascular inflammation observed in VBD does not result from isolated abnormalities of a single immune cell population. Instead, it represents a coordinated interaction among multiple immune compartments that establishes a self-amplifying inflammatory network.

Neutrophils activate macrophages and Th17 cells through NET formation; macrophages promote VSMC phenotypic switching and vascular matrix degradation through THBS1 signaling; Th17 cells recruit additional neutrophils through IL-17A secretion; CD8^+^ T cells exert direct cytotoxic effects against vascular wall cells under HLA-B51-mediated antigen presentation; and impaired Treg function removes critical restraints on these inflammatory effector pathways (9, 24, 29).

This integrated network involving both innate and adaptive immunity provides the immunological basis for persistent vascular inflammation, progressive vascular destruction, and recurrent disease activity in VBD. Among these interconnected pathways, the THBS1–macrophage axis and the NETs–IL-17A inflammatory circuit may represent key regulatory nodes and candidate targets for precision therapeutic intervention.

## 5 Key inflammatory mediators and signaling pathways

### 5.1 The central pathogenic role of TNF-α

TNF-α occupies a central position within the inflammatory network of Behçet disease, a concept strongly supported by the clinical efficacy of TNF-α inhibitors in refractory VBD. A multicenter observational study involving 37 patients with VBD demonstrated that infliximab achieved a clinical response rate of 73% in patients with refractory vascular manifestations, with nearly a 50% reduction in recurrent vascular events compared with conventional immunosuppressive therapy (38). A 2025 systematic review further confirmed that infliximab treatment promotes aneurysm regression and improves radiological manifestations of vascular inflammation (39).

Mechanistically, the role of TNF-α extends far beyond a conventional pro-inflammatory cytokine. TNF-α enhances endothelial expression of adhesion molecules, including intercellular adhesion molecule-1 (ICAM-1) and vascular cell adhesion molecule-1 (VCAM-1), thereby promoting leukocyte adhesion and transendothelial migration. It activates macrophages to release MMPs, directly contributing to extracellular matrix degradation within the vascular wall. In addition, TNF-α induces VSMC phenotypic switching, weakening vascular structural integrity, and enhances neutrophil activation to amplify NET formation (1, 5).

Therefore, TNF-α acts as a central integrator connecting multiple pathological processes involved in VBD, including immune-cell recruitment, inflammatory amplification, vascular remodeling, and tissue destruction. This mechanistic position explains why TNF-α blockade has achieved substantial clinical benefits. However, inhibition of TNF-α does not completely prevent recurrent vascular inflammation, suggesting the existence of TNF-α-independent inflammatory pathways, including IL-6- and IL-1β-mediated signaling cascades (40, 41).

### 5.2 Matrix metalloproteinases and extracellular matrix degradation

Degradation of vascular extracellular matrix (ECM) represents the immediate structural basis underlying pseudoaneurysm formation, with matrix metalloproteinases (MMPs) serving as the major executors of this process.

MMP-2 and MMP-9 are markedly upregulated in affected vascular tissues of VBD patients. MMP-2 primarily degrades type IV collagen within basement membranes, whereas MMP-9 targets interstitial type I and type III collagen as well as elastin fibers. Destruction of elastic fibers results in loss of vascular recoil capacity, while collagen degradation compromises tensile strength, collectively rendering the vascular wall vulnerable to progressive dilation under hemodynamic stress and ultimately leading to aneurysm or pseudoaneurysm formation (5, 42).

At least two major cellular sources contribute to MMP production in VBD: activated macrophages and phenotypically transformed VSMCs. THBS1”high macrophages promote VSMC transition from a contractile to a synthetic phenotype through paracrine THBS1 signaling, thereby generating MMP-producing VSMCs (9).

More broadly, ECM degradation and inflammation form a self-perpetuating feedback loop in aneurysm biology. Matrix fragments generated during ECM breakdown function as damage-associated molecular patterns (DAMPs), further activating immune cells and inducing additional MMP release, thereby sustaining progressive vascular destruction (42, 43).

The YAP/TAZ pathway has also emerged as an important regulator linking mechanical stress to vascular remodeling. Specific deletion of YAP/TAZ signaling in VSMCs induces aortic aneurysm formation in mice, suggesting that mechanotransduction pathways are critically involved in maintaining ECM homeostasis and vascular integrity (44).

### 5.3 Cooperative inflammatory mediator networks

Beyond TNF-α and MMPs, multiple inflammatory mediators participate cooperatively in vascular injury associated with VBD.

IL-17A, primarily produced by Th17 cells, promotes neutrophil recruitment and activation, as described above. IL-1β, generated downstream of inflammasome activation, appears to play a less dominant role in BD compared with autoinflammatory diseases such as familial Mediterranean fever; however, the clinical efficacy of IL-1-targeted biologics in selected refractory BD patients suggests that IL-1 signaling represents an alternative therapeutic pathway beyond TNF-α inhibition (45).

Chemokine networks are essential for immune-cell trafficking into vascular tissues. Multiple chemokines and their receptors are dysregulated in BD patients, providing the molecular basis for inflammatory cell recruitment and accumulation within vascular lesions (46).

The JAK/STAT signaling pathway functions as a major intracellular hub downstream of multiple cytokine receptors and has been reported to be activated in BD (47). A 2026 systematic review confirmed the therapeutic efficacy and safety profile of JAK inhibitors in BD syndromes, further supporting the pathogenic relevance of this pathway (48).

Bioinformatic analyses have demonstrated abnormal activation of the IL-23/IL-17 axis in BD, providing a rationale for targeting upstream regulators of IL-17-mediated inflammation (49).

Additional inflammatory and metabolic pathways may also contribute to vascular injury. Elevated homocysteine levels have been associated with endothelial dysfunction and vascular involvement in VBD (50). Asprosin, a newly identified inflammation–metabolism regulatory molecule, correlates closely with disease activity and vascular involvement and may represent a potential biomarker for future disease monitoring (51). Endothelial dysfunction and complement activation have been independently associated with BD activity (52). Furthermore, lipidomic remodeling has been linked to vascular involvement in VBD, suggesting that metabolic dysregulation contributes to vascular disease progression (53).

## 6 Pathological mechanisms underlying vascular wall destruction

### 6.1 The vasa vasorum as an initial target of inflammatory attack

The vascular wall is not an immunologically privileged structure. Instead, it possesses an intrinsic microvascular network, the vasa vasorum, which supplies oxygen and nutrients to the adventitia and outer media.

Increasing evidence supports the concept that vascular inflammation in VBD may originate from the vasa vasorum rather than the intimal layer (54, 55). The adventitial vasa vasorum represents the first interface through which circulating immune cells interact with the vascular wall. Activated neutrophils, monocytes, and T cells infiltrate through vasa vasorum endothelial barriers and initiate inflammatory cascades within the adventitia and outer media, subsequently extending toward deeper vascular layers.

Inflammation of the vasa vasorum may induce ischemic injury within the vascular media, further compromising structural integrity. This outside-in inflammatory progression pattern contrasts with the classical inside-out model observed in atherosclerosis and may explain several distinctive features of VBD-associated aneurysms, including prominent adventitial thickening, perivascular soft tissue inflammation, and full-thickness vascular involvement rather than isolated intimal pathology.

From a broader aneurysm biology perspective, abnormal expansion and dysfunction of the vasa vasorum have also been reported in aortic aneurysms and dissections, suggesting that this microvascular network may represent a common initiating factor across multiple vascular diseases (55, 56). This evidence is borrowed from non-VBD aneurysm models and provides mechanistic plausibility rather than direct proof for VBD.

### 6.2 Inflammatory destruction of the arterial media

**Figure 1:**
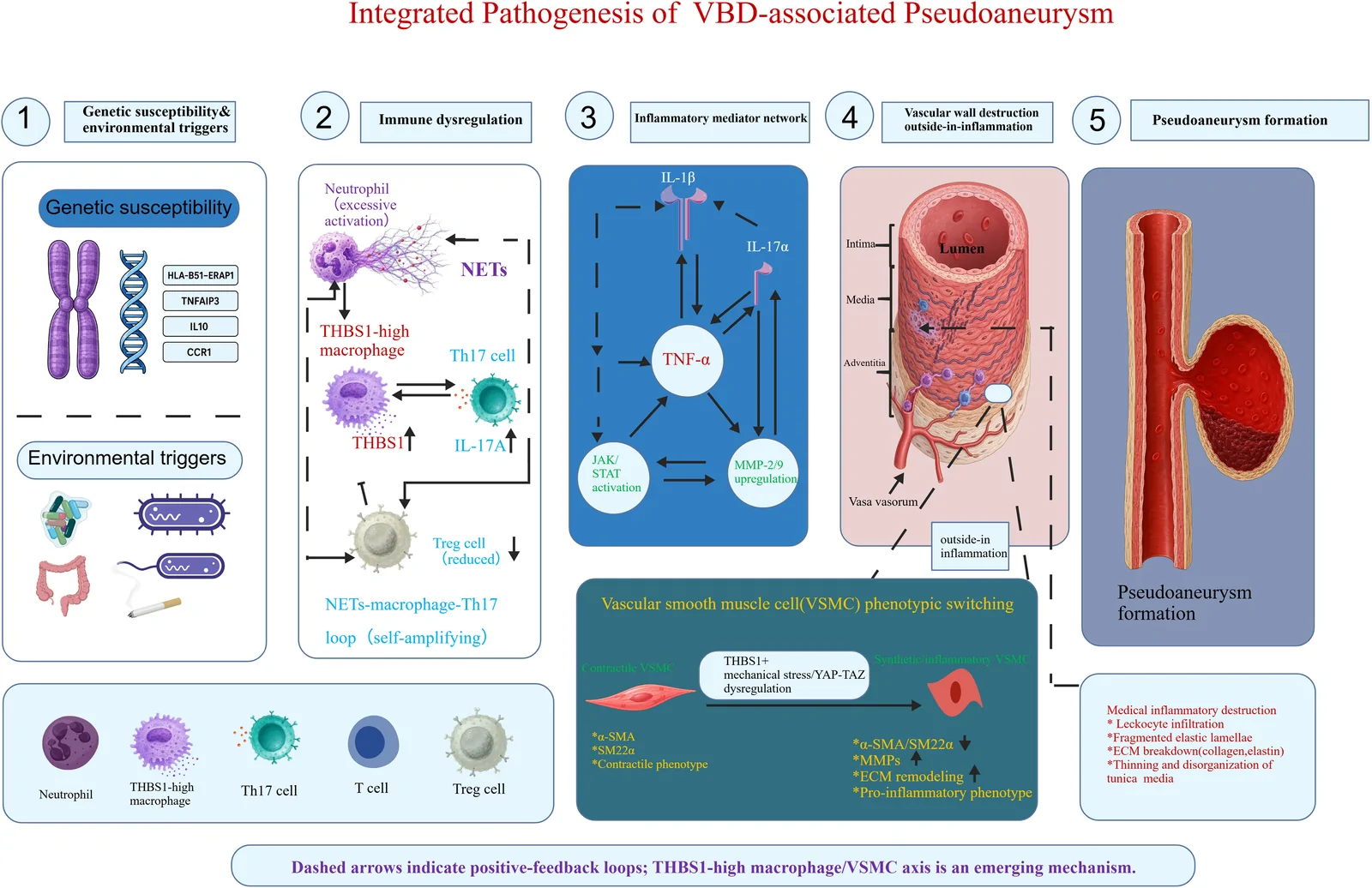
Integrated pathogenesis of VBD-associated pseudoaneurysm. The diagram reads left-to-right as a five-module cascade: (1) genetic susceptibility (HLA-B51/ERAP1; TNFAIP3, IL10, CCR1) and environmental triggers (microbial infection, mucosal dysbiosis, environmental stress); (2) immune dysregulation, in which activated neutrophils release neutrophil extracellular traps (NETs) that activate THBS1-high macrophages, with aberrant Th17 responses and reduced Treg activity forming a NETs–macrophage–Th17 positive-feedback loop; (3) an inflammatory mediator network centered on TNF-α that cooperates with IL-1β, IL-17A, JAK/STAT signaling, MMP-2/9 upregulation, and leukocyte recruitment; (4) vascular wall destruction, where inflammation propagates from the adventitial vasa vasorum toward the media in an outside-in manner, causing leukocyte infiltration, elastic lamellar fragmentation, MMP-mediated extracellular matrix degradation, medial thinning, and loss of wall integrity; and (5) pseudoaneurysm formation with full-thickness wall disruption, a narrow communicating neck, extraluminal sac, mural thrombus, and risk of rupture. The dashed inset box below module 4 details vascular smooth muscle cell (VSMC) phenotypic switching from a contractile to a synthetic/inflammatory phenotype, driven by THBS1 signaling, TNF-α/IL-1β, inflammatory and mechanical stress, and YAP/TAZ dysregulation, with the resulting synthetic VSMCs feeding back to medial ECM degradation. Solid arrows indicate established directional relationships; dashed arrows indicate proposed positive-feedback loops. The THBS1-high macrophage/VSMC phenotypic-switch axis is an emerging mechanism, and YAP/TAZ-related regulation is partly extrapolated from non-VBD aneurysm models (see Section 8.7). Created by the authors using licensed graphical elements from BioGDP.com under a Standard Academic License (Agreement No. GDP2026Z77734).

The arterial media represents the major structural layer responsible for resisting hemodynamic stress and consists of concentric layers of elastic lamellae and VSMCs (**Figure 1**).

In VBD, inflammatory cells infiltrating from the adventitia through the vasa vasorum release MMP-2, MMP-9, and elastase, leading to progressive degradation of elastic lamellae within the media. Loss of elastic fibers reduces vascular recoil capacity, whereas inflammatory mediators simultaneously induce VSMC apoptosis or phenotypic switching. The depletion of contractile VSMCs further compromises active regulation of vascular wall tension (5, 9).

Once elastic fiber disruption and VSMC loss reach a critical threshold, localized vascular dilation occurs under continuous hemodynamic stress. Progressive dilation increases mechanical loading on the vascular wall, activating YAP/TAZ mechanotransduction pathways in VSMCs. Activated YAP/TAZ further promotes phenotypic switching and MMP production, establishing a vicious cycle of vascular expansion → mechanical stress → VSMC reprogramming → ECM degradation → further expansion (44).

The discovery that BAF60c regulates VSMC function through epigenetic mechanisms and protects against abdominal aortic aneurysm formation provides additional evidence that epigenetic regulation is essential for maintaining VSMC homeostasis (57).

When inflammatory destruction extends through all vascular layers and breaches the adventitia, blood extravasation occurs, resulting in pseudoaneurysm formation. At this stage, the complete pathogenic transition from molecular immune activation to macroscopic vascular failure becomes evident.

### 6.3 Vascular smooth muscle cell phenotypic switching

VSMC phenotypic switching represents the critical bridge connecting immune inflammation with structural vascular destruction (**Figure 1**).

Under physiological conditions, adult arterial VSMCs maintain a contractile phenotype characterized by expression of contractile markers such as α-SMA and SM22α, enabling regulation of vascular tone and maintenance of vessel integrity. Under inflammatory stimulation, VSMCs undergo transition from a contractile phenotype toward a synthetic phenotype. Synthetic VSMCs lose contractile function but acquire enhanced proliferative, migratory, and MMP-secretory capabilities.

A 2025 BMC Medicine study provided single-cell evidence supporting this process in VBD, although this remains a single-center finding awaiting replication. THBS1 secreted by THBS1”high macrophages was identified as a key signal driving VSMC phenotypic switching (9).

Following phenotypic transformation, VSMCs release MMP-2 and MMP-9, directly degrading their surrounding ECM framework. Their enhanced proliferation and migration further contribute to vascular wall remodeling and aneurysmal transformation.

The YAP/TAZ pathway functions as a mechanosensitive amplifier during this process. Progressive vascular dilation increases mechanical stress, activates YAP/TAZ signaling, and subsequently promotes additional VSMC phenotypic switching and MMP secretion (44).

Collectively, a series of interconnected feed-forward loops characterize VBD-associated pseudoaneurysm progression: inflammation drives VSMC phenotypic switching → phenotypic switching accelerates ECM degradation → ECM degradation promotes vascular dilation → mechanical stress reinforces VSMC reprogramming.

This multilayered pathological network provides the cellular basis for progressive pseudoaneurysm expansion and the high recurrence rate after intervention. Epigenetic regulators, including BAF60c, may represent promising therapeutic targets for disrupting this pathological circuit (57).

## 7 Animal models and mechanistic validation

Animal models are essential for establishing causal relationships between molecular mechanisms and disease phenotypes. Current experimental models of Behçet disease (BD) mainly include herpes simplex virus (HSV)-induced inflammatory models and autoimmune-based models. However, a dedicated animal model that faithfully reproduces vascular Behçet’s disease (VBD)-associated pseudoaneurysms remains unavailable.

The HSV-induced BD-like inflammatory model represents the most widely used experimental system. In this model, inoculation of HSV into the auricular skin of susceptible mouse strains induces BD-like manifestations, including oral ulceration, mucocutaneous lesions, and ocular inflammation. This model has provided valuable insights into inflammatory mechanisms underlying BD. For example, vitamin D3 supplementation has been shown to ameliorate HSV-induced BD-like inflammation (58). MicroRNA-21 has been identified as an important regulatory molecule in HSV-triggered inflammatory responses (59). Furthermore, IL-2/IL-2 antibody immune complexes can enhance regulatory T-cell (Treg) activity and alleviate HSV-induced inflammation (60). The CCR1–CCL3 chemokine axis has also been implicated in leukocyte recruitment in HSV-induced BD models, and blockade of CCL3 signaling reduces inflammatory responses (61).

The potential role of HSV in BD pathogenesis itself remains an active area of investigation. The molecular mimicry hypothesis proposes that structural similarities between HSV-derived antigens and host tissue antigens may trigger autoimmune responses through cross-reactive immune activation (62). In addition, bioinformatics-based drug screening combined with experimental validation in mouse models has provided novel therapeutic insights into BD treatment strategies (63).

Despite these advances, current animal models have substantial limitations. HSV-induced models primarily reproduce mucosal and ocular inflammation but rarely develop vascular lesions, particularly pseudoaneurysms, which represent the defining clinical manifestation of VBD. Similarly, although HLA-B51 transgenic mouse models have been reported, they have not demonstrated consistent vascular phenotypes. This lack of a vascular phenotype–relevant model represents a major obstacle to *in vivo* validation of mechanisms driving VBD-associated pseudoaneurysm formation.

Future strategies may include combining immune activation approaches with established aneurysm-prone backgrounds, such as apolipoprotein E (ApoE)-deficient or LDL receptor (LDLR)-deficient mice, together with vascular inflammatory induction approaches (for example, perivascular inflammatory stimulation) to generate inflammation-driven aneurysm models. Alternatively, humanized mouse models integrating HLA-B51 transgenic expression with human immune-cell reconstruction may provide a more physiologically relevant platform for studying VBD immunopathology.

Only when experimental models can reliably reproduce the complete pathological sequence—from immune activation and vascular inflammation to vascular wall destruction and pseudoaneurysm formation—will current mechanistic hypotheses achieve definitive causal validation.

To provide historical balance, it is worth noting that mechanistic understanding of BD has accumulated over several decades: early studies established neutrophil hyperreactivity and the HLA-B51 association as foundational observations, while HSV-induced animal models introduced in the 2000s have long been used to probe inflammatory pathways (34, 47, 58, 62). The more recent single-cell and multi-omics era has refined, rather than replaced, these earlier frameworks. Previous reviews of vascular BD have focused predominantly on clinical manifestations, surgical management, and epidemiology rather than on pseudoaneurysm-specific mechanisms (3, 4, 14); the present review attempts to address this mechanistic niche, while acknowledging that its proposed framework rests on evidence of varying directness (see Section 8.7).

## 8 An integrated multilevel model of VBD pseudoaneurysm pathogenesis

Based on current evidence, the pathogenesis of VBD-associated pseudoaneurysms can be integrated into a multilevel pathogenic cascade. The integrated model is summarized schematically in **Figure 1**.

### 8.1 Genetic susceptibility: establishing an inflammatory-prone background

The first layer represents genetic predisposition. The HLA-B51/ERAP1 axis alters antigen peptide processing and presentation, while variants in TNFAIP3, IL10, CCR1, and other immune-regulatory genes establish a lower threshold for immune activation, thereby reducing the ability of the host immune system to restrain excessive inflammatory responses.

### 8.2 Environmental triggers: initiating immune dysregulation

The second layer involves environmental triggers. Factors such as HSV infection and microbiome perturbation may disrupt immune homeostasis through molecular mimicry or innate immune activation, initiating downstream inflammatory cascades in genetically susceptible individuals (1, 62).

### 8.3 Immune-cell amplification: formation of self-sustaining inflammatory circuits

The third layer involves coordinated amplification among immune-cell populations. Hyperresponsive neutrophils generate excessive NETs, which promote macrophage activation toward the THBS1”high macrophage phenotype and enhance Th17-cell responses. Th17 cells produce IL-17A, which recruits and activates additional neutrophils. Meanwhile, HLA-B51-restricted CD8^+^ T-cell responses may induce cytotoxic injury against vascular wall cells. Impaired Treg function removes critical immune restraints, allowing innate and adaptive immune responses to establish a self-sustaining feed-forward inflammatory network (9, 17, 24, 29).

### 8.4 Inflammatory mediator networks: propagation of vascular injury signals

The fourth layer involves systemic propagation of inflammatory mediators. TNF-α functions as a central inflammatory driver connecting multiple pathological processes. MMPs execute extracellular matrix degradation, while IL-17A, IL-1β, chemokine networks, and JAK/STAT signaling pathways act cooperatively to establish a highly interconnected inflammatory signaling network (1, 5, 48).

### 8.5 Structural vascular destruction: terminal pathological outcome

The fifth layer represents irreversible vascular wall destruction. Inflammation propagates from the adventitial vasa vasorum toward deeper vascular layers through an outside-in mechanism. MMP-mediated degradation causes disruption of medial elastic fibers, while THBS1 signaling drives VSMC phenotypic switching. Mechanical stress generated by progressive vascular dilation activates YAP/TAZ mechanotransduction pathways, converting biomechanical signals into molecular programs that further promote VSMC reprogramming and matrix degradation. These interconnected feedback loops ultimately result in full-thickness vascular wall disruption and pseudoaneurysm formation (9, 44, 54).

### 8.6 Therapeutic implications and translational challenges

This multilevel pathogenic framework provides important therapeutic implications. Repairing vascular structural damage alone through surgery or endovascular intervention addresses only the terminal consequence of disease without eliminating the upstream immunological drivers, thereby explaining the high recurrence rate observed after intervention.

An optimal therapeutic strategy should therefore target multiple pathogenic layers simultaneously. TNF-α inhibitors may suppress central inflammatory drivers, whereas immunosuppressive therapies may restore immune-cell balance. Definitive vascular repair should ideally be performed after adequate inflammatory control, followed by sustained postoperative immunomodulation to prevent reactivation of inflammatory circuits.

Furthermore, precision therapies targeting key regulatory nodes, particularly the THBS1–macrophage axis and the NETs–IL-17A inflammatory circuit, may represent promising approaches for preventing pseudoaneurysm progression and improving long-term outcomes in VBD.

Current management of VBD-associated pseudoaneurysms follows a staged principle: acute control of vascular inflammation with corticosteroids and conventional immunosuppressants (e.g., cyclophosphamide, azathioprine, ciclosporin), followed by early introduction of biologic therapy—principally TNF-α inhibitors (infliximab, adalimumab)—for refractory or relapsing disease, with surgical or endovascular repair deferred until inflammation is adequately controlled (38, 39, 64). The 2018 update of the EULAR recommendations for the management of Behçet syndrome formalized this immunosuppression-first approach for vascular involvement (64). Colchicine and, in selected cases, interferon-α retain adjunctive roles, while JAK inhibitors have emerged as a promising option supported by recent systematic review evidence (48).

Despite these advances, current therapies are nonspecific and do not directly target the pathogenic nodes identified in this review. Two candidate axes merit dedicated translational consideration. The THBS1–macrophage axis is mechanistically attractive because THBS1 signaling links macrophage activation to VSMC phenotypic switching (9); however, no THBS1-targeted therapy currently exists for clinical use. THBS1 exerts pleiotropic, context-dependent effects through multiple receptors (including CD36, CD47, and integrins), and systemic blockade risks impairing wound healing, anti-angiogenic homeostasis, and tumor surveillance. Development of pathway-selective modulators or cell-type-targeted delivery would be required before clinical translation; at present, the most pragmatic approach to modulating this axis indirectly is early, aggressive control of macrophage-driven inflammation with TNF-α inhibitors or JAK inhibitors.

The NETs–IL-17A circuit is similarly plausible but unvalidated in VBD. IL-17A biologics (secukinumab, ixekizumab) and IL-23/IL-12 inhibitors (ustekinumab) are approved for other Th17-mediated diseases and are biologically plausible candidates, but clinical evidence in VBD-associated pseudoaneurysm remains limited and anecdotal, and concerns exist regarding potential exacerbation of Crohn-like intestinal disease with IL-17 blockade. Anti-NET strategies—such as recombinant DNase, PAD4 inhibitors, and anti-histone antibodies—are preclinical and untested in VBD, and netosis-targeted intervention carries infection-risk trade-offs given the antimicrobial role of NETs.

Translational challenges and biomarker requirements. Several barriers temper near-term precision-medicine aspirations: (i) the absence of validated biomarkers that stratify patients by dominant pathogenic axis (THBS1-macrophage- vs NETs-IL-17-dominant); (ii) the lack of pseudoaneurysm-relevant animal models for preclinical testing (see Section 7); (iii) the rarity and clinical heterogeneity of VBD, which impede adequately powered trials; and (iv) the risk that intensification of immunosuppression may complicate perioperative management. Realization of precision intervention will therefore require prospective biomarker-driven cohort studies and, ultimately, mechanism-stratified trials rather than empirical escalation.

### 8.7 Strength of evidence, methodological limitations, and controversies

The evidence synthesized in this review is heterogeneous in directness and certainty, and several limitations should be acknowledged explicitly.

Strength of evidence and source stratification. The most direct evidence comes from human VBD aneurysmal tissue analyses, but these are limited to a small number of single-center studies with modest sample sizes. The single-cell identification of THBS1”high macrophages (9), for example, derives from one exploratory cohort and has not yet been independently replicated; its conclusions should therefore be treated as hypothesis-generating. Findings from non-vascular BD tissues (e.g., uveitis iris transcriptomics (35)) and from peripheral blood are informative but indirect, as they may not reflect the vascular wall microenvironment. Evidence borrowed from other aneurysm models—abdominal aortic aneurysm and aortic dissection (42, 44, 55)—provides mechanistic plausibility but assumes conservation across distinct disease entities. Bioinformatic predictions (e.g., IL-23/IL-17 axis activation (49)) require experimental confirmation. **Table 1** indicates the source type and key limitations for each mechanism.

Methodological limitations. Most cited studies are observational, cross-sectional, or transcriptomic, and therefore establish association rather than causation. Sample sizes are frequently small, tissue acquisition is subject to selection bias (resected aneurysms represent end-stage disease), and disease-activity confounding is seldom fully addressed. Single-cell and spatial omics atlases of VBD vascular lesions remain scarce (10).

Replication status and controversies. Few molecular findings in VBD have undergone independent replication, and some remain contested. The relative contribution of IL-1β is uncertain: it appears less dominant in BD than in classic autoinflammatory diseases, yet IL-1-directed biologics benefit selected refractory patients (45). The role of anticoagulation in BD-associated thrombosis remains debated, with growing support for an immunosuppression-rather-than-anticoagulation paradigm. Whether the outside-in pattern of inflammation is unique to VBD or shared with other non-atherosclerotic aneurysms also awaits clarification. These gaps underscore that the integrated model proposed here is a working framework rather than a definitive mechanistic account.

## 9 Summary and future perspectives

The pathogenesis of VBD-associated pseudoaneurysms represents a multilevel pathological cascade extending from genetic susceptibility to progressive vascular wall destruction. The HLA-B51/ERAP1 axis establishes an inherited susceptibility background, while environmental triggers disrupt immune homeostasis and initiate inflammatory activation. Subsequently, a self-amplifying inflammatory network centered on the NET–macrophage–Th17 axis drives persistent immune activation. TNF-α acts as a central inflammatory hub connecting multiple pathological processes, whereas MMPs execute extracellular matrix degradation. In parallel, inflammation originating from the vasa vasorum propagates in an outside-in manner, resulting in medial elastic fiber disruption, VSMC phenotypic switching, and ultimately full-thickness vascular wall rupture leading to pseudoaneurysm formation.

The identification of THBS1”high macrophages in a 2025 BMC Medicine study represents a potentially important advance in this field, providing single-cell-level evidence linking a pathogenic immune-cell subset, VSMC phenotypic transition, and aneurysm development; however, as a single-center finding, it requires independent replication before being considered definitive (9). However, substantial knowledge gaps remain.

From the perspective of experimental modeling, current HSV-induced models and HLA-B51 transgenic models fail to consistently reproduce vascular phenotypes, severely limiting *in vivo* validation of proposed mechanisms. From a genetic perspective, dedicated GWAS studies focusing specifically on pseudoaneurysm phenotypes are still lacking. Consequently, how genetic variation determines whether BD patients develop vascular involvement, and whether such involvement manifests as pseudoaneurysm or other vascular complications, remains largely unresolved. Furthermore, despite rapid advances in single-cell and spatial omics technologies, comprehensive molecular atlases of VBD vascular lesions remain extremely limited. A recent omics-based review published in 2025 emphasized the urgent need to address this critical knowledge gap (10).

Future investigations should prioritize several key directions. First, development of robust animal models that faithfully reproduce VBD-associated pseudoaneurysm formation is essential. Humanized HLA-B51 transgenic models combined with vascular inflammatory induction strategies may provide a promising experimental platform. Second, dedicated GWAS and whole-exome sequencing studies in VBD vascular lesions are required to identify genetic determinants specifically associated with pseudoaneurysm susceptibility. Third, integration of single-cell RNA sequencing and spatial transcriptomics should be employed to generate comprehensive cellular and molecular maps of affected vascular tissues and identify additional pathogenic cell populations analogous to THBS1”high macrophages.

Moreover, therapeutic strategies targeting key inflammatory circuits, particularly the THBS1–macrophage axis and the NETs–IL-17A inflammatory loop, warrant further investigation as potential approaches to overcome current treatment limitations. Ultimately, integrating multi-omics datasets with clinical parameters may enable the development of predictive models for vascular involvement risk and facilitate translation from mechanistic discoveries to precision medicine.

Research into VBD-associated pseudoaneurysm pathogenesis is undergoing a fundamental transition from descriptive characterization toward molecularly defined mechanisms. This paradigm shift may provide new opportunities to address one of the most challenging vascular manifestations of Behçet disease.

## 10 Key points

- Vascular Behçet’s disease (VBD)-associated pseudoaneurysms represent a unique inflammatory vascular disorder driven by transmural immune-mediated destruction rather than atherosclerotic degeneration.
- A multilevel pathogenic cascade involving genetic susceptibility (HLA-B51/ERAP1 axis), immune-cell dysregulation, inflammatory mediator networks, and vascular wall remodeling underlies pseudoaneurysm formation and rupture.
- Emerging single-cell studies have identified THBS1”high macrophages as a pathogenic immune-cell population that may link macrophage activation, vascular smooth muscle cell phenotypic switching, and aneurysm development; this finding awaits independent replication.
- Neutrophil extracellular traps (NETs), Th17 responses, and IL-17A-mediated inflammatory circuits establish self-amplifying immune networks that sustain vascular inflammation and promote progressive vascular destruction.
- Future advances require vascular phenotype–relevant animal models, pseudoaneurysm-focused genetic studies, validated biomarkers for mechanism stratification, and multi-omics-guided precision therapies targeting key inflammatory circuits.

## Glossary

BD: Behçet disease
VBD: Vascular Behçet’s disease
MHC-I: Major histocompatibility complex class I
ERAP1: Endoplasmic reticulum aminopeptidase 1
GWAS: Genome-wide association study
HSV: Herpes simplex virus
NETs: Neutrophil extracellular traps
NETosis: Neutrophil extracellular trap formation
MPO: Myeloperoxidase
VSMCs: Vascular smooth muscle cells
THBS1: Thrombospondin-1
ECM: Extracellular matrix
MMPs: Matrix metalloproteinases
DAMPs: Damage-associated molecular patterns
ICAM-1: Intercellular adhesion molecule-1
VCAM-1: Vascular cell adhesion molecule-1
TNF-α: Tumor necrosis factor-α
IL-1β: Interleukin-1β
IL-10: Interleukin-10
IL-17A: Interleukin-17A
IL-23: Interleukin-23
JAK/STAT: Janus kinase/signal transducer and activator of transcription
Treg: Regulatory T cell
Th17: T helper 17 cell
CD8+ T cell: Cluster of differentiation 8-positive T cell
CCR1: C-C chemokine receptor type 1
CCL3: C-C motif chemokine ligand 3
MCP-1: Monocyte chemoattractant protein-1
ACE: Angiotensin-converting enzyme
YAP/TAZ: Yes-associated protein/transcriptional coactivator with PDZ-binding motif
α-SMA: Alpha-smooth muscle actin
SM22α: Smooth muscle protein 22-alpha
BAF60c: Brahma-associated factor 60c
ApoE: Apolipoprotein E
LDLR: Low-density lipoprotein receptor
RNA-seq: RNA sequencing
scRNA-seq: Single-cell RNA sequencing
WES: Whole-exome sequencing
C1q: Complement component 1q
SHP2: Src homology region 2 domain-containing phosphatase-2
LCK: Lymphocyte-specific protein tyrosine kinase
EULAR: European Alliance of Associations for Rheumatology
MHC-I-opathy: MHC class I-associated autoinflammatory disease
DVT: Deep vein thrombosis.
Level: Core linkLevel 1 –
Genetic susceptibility: HLA-B51/ERAP1 axis
Environmental triggers: HSV infection/microbiota dysbiosis
Immune synergistic amplification: NETs–macrophage–Th17 positive feedback loop
Inflammatory mediator dissemination: TNF-α
Vascular wall destruction: Vasa vasorum inflammation → medial disintegration → VSMC phenotypic switching → pseudoaneurysm
